# Effect of Metronidazole on Concentrations of Vaginal Bacteria Associated with Risk of HIV Acquisition

**DOI:** 10.21203/rs.3.rs-4219764/v1

**Published:** 2024-04-11

**Authors:** D.J. Valint, Tina L. Fiedler, Congzhou Liu, Sujatha Srinivasan, David N. Fredricks

**Affiliations:** 1 Vaccine and Infectious Disease Division, Fred Hutchinson Cancer Center, Seattle, WA, USA;; 2 Department of Medicine, University of Washington, Seattle, WA, USA

## Abstract

Several bacterial vaginosis (BV)-associated bacteria have been associated with elevated risk of HIV acquisition, however susceptibility of these bacteria to antibiotics is poorly understood. Vaginal samples were collected from 22 persons daily for two weeks following BV diagnosis. Metronidazole treatment was prescribed for 5–7 days. Changes in bacterial concentrations were measured with taxon-specific 16S rRNA gene quantitative PCR (qPCR) assays. A culture-based antimicrobial assay confirmed presence of antibiotics in vaginal swab samples. Bacterial DNA concentrations decreased during antibiotic administration for all thirteen bacterial taxa tested. Comparison of bacterial DNA concentrations in samples before administration of antibiotics to samples taken on the last day of antimicrobial assay-confirmed antibiotic presence showed a 2.3–4.5 log10-fold decrease across all taxa. Concentrations were frequently reduced to the qPCR assay’s limit of detection, suggesting eradication of bacteria. Mean clearance time varied across taxa (1.2–8.6 days), with several bacteria (e.g., *Gemella asaccharolytica*, *Sneathia* spp., *Eggerthella*-like sp.) taking >7 days to suppress. Metronidazole reduces quantities of bacterial taxa associated with increased HIV acquisition risk. Eradication of high-risk vaginal bacteria using metronidazole is one promising avenue for reducing HIV acquisition risk. A 5–7-day treatment course may not be sufficient to suppress all bacteria.

## Summary

Metronidazole significantly decreases concentrations of numerous vaginal bacteria associated with increased HIV infection risk. The time course of bacterial eradication was variable and depended on initial bacterial concentrations. Metronidazole treatment offers one potential avenue to decrease HIV infection risk.

## Background

Bacterial vaginosis (BV) is a gynecologic condition characterized by a shift in the vaginal microbiota in which the healthy *Lactobacillus*-dominated community is replaced by a complex and heterogeneous consortium of anaerobic bacteria [Fredricks 2005, Srinivasan 2010, Srinivasan 2012, Ravel 2013]. This shift in the microbial community is associated with several adverse sequelae, including an increased risk of sexually transmitted infections such as with human immunodeficiency virus (HIV) [Martin 1999, Cohen 2012]. A broad array of vaginal bacterial species has been associated with elevated risk of subsequent HIV acquisition, including *Prevotella* spp., *Sneathia* spp., *Parvimonas* spp., *Gemella asaccharolytica*, *Mycoplasma hominis*, *Porphyromonas* species Type 1, *Eggerthella*-like vaginal bacterial species Type 1, *Megasphaera* spp., *Amygdalobacter indicium* (previously BV-associated bacterium 2 [Srinivasan 2023]), *Peptoniphilus lacrimalis* and Vaginal TM7 [McClelland 2018, Gosmann 2017, Srinivasan 2018, 2019]. Bacterial associations with HIV risk may be due to increased genital inflammation caused by bacteria [McClelland 2018, Srinivasan 2018, Srinivasan 2019, Anahtar 2015], disruption of the cervicovaginal mucus layer by bacterial sialidases and mucinases [McGregor 1994, Cauci 2011], or other factors. Regardless, identifying an effective intervention that can target these bacteria would allow for the assessment of the direct impact of bacteria on HIV acquisition and would provide a critical step towards devising additional preventive measures to mitigate HIV infection risk if bacteria play a causal role.

Current CDC guidelines recommend metronidazole as first-line treatment for BV, though clindamycin, secnidazole, and tinidazole may also be used [Weir 2023, Workowski 2021]. Metronidazole is often the preferred option due to its efficacy and low cost [Schwebke 2007, Schmitt 1992, Sobel 1997] and administered as a topically applied vaginal gel or orally ingested pills. Metronidazole is an antibiotic prodrug activated via reduction under anaerobic conditions and targets many anaerobic bacteria present in BV. Metronidazole treatment can have a dramatic effect on the vaginal bacterial community, resulting in a widespread reduction in bacterial concentrations across the diverse community of BV-associated bacteria [Fredricks 2009]. For some vaginal bacterial taxa such as *Gardnerella* spp. and *Fannyhessea vaginae*, metronidazole was effective in reducing the concentrations but not eradicating these bacteria [Bradshaw 2006, Mayer 2015].

For many of the HIV risk-associated vaginal bacterial species, susceptibility to antibiotics is poorly documented. Many of these organisms are fastidious making direct measurements of *in vitro* antibiotic susceptibility difficult. Moreover, there is a paucity of *in vivo*, longitudinal studies of the vaginal microbiota that utilize quantitative PCR techniques to assess absolute changes in concentrations of vaginal bacterial species with antibiotic therapy. Based on an NCBI PubMed search with the search terms “(vaginal) AND ((microbiome) OR (microbiota)) AND (metronidazole) AND ((quantitative PCR) OR (qPCR))”, as of October 2023, only seven studies reported longitudinal, *in vivo* qPCR data describing the shifts in vaginal bacterial concentrations over the course of metronidazole treatment [Armstrong 2022(a), Armstrong 2022(b), van de Wijgert 2020, Balkus 2017, Ahrens 2020, Srinivasan 2010, Mayer 2015]. Of these, only four included data on the concentrations of at least one bacterial taxon associated with HIV-risk [Armstrong 2022(a), Balkus 2017, Srinivasan 2010, Mayer 2015], and only two collected daily vaginal samples subjected to qPCR [Srinivasan 2010, Mayer 2015]. While the findings of Srinivasan et al. (2010) and Mayer et al. (2015) help to elucidate the dynamic behavior of the vaginal bacterial community during and following antibiotic treatment for BV, both studies included a relatively small number of participants with BV and neither study specifically sought to clarify how HIV-risk-associated bacterial taxa respond to metronidazole treatment.

To fill this knowledge gap, we leveraged data from a longitudinal study of persons with BV treated with metronidazole to determine how concentrations of vaginal bacteria change *in vivo* in response to antibiotic therapy, circumventing the need for laboratory cultivation to assess susceptibility. We targeted 13 vaginal bacterial species previously linked to elevated HIV infection risk and measured concentrations of bacterial DNA by qPCR in a longitudinal sample set of vaginal swabs collected daily for a two-week period following BV diagnosis, capturing the entire duration of treatment and approximately one week following cessation. Our immediate goal was to determine whether metronidazole leads to eradication of high-risk vaginal bacteria, with an ultimate goal of identifying an intervention that could be studied to deplete these bacteria and assess impact on HIV infection risk.

## Methods

### Study Population, Treatment and Sampling

Between October 2012 and June 2016, persons with BV were enrolled in a study of the vaginal microbiota conducted by the University of Washington and the Fred Hutch Cancer Center in Seattle, WA. Protocols were approved by the Fred Hutch Institutional Review Board (IRB Protocol #7683) and all study participants provided written informed consent. For each participant with BV, antibiotic treatment with metronidazole was prescribed either as 5 g of vaginally applied gel (with 37.5 mg of metronidazole) daily for 5 days, or orally ingested pills of 500 mg twice daily for 7 days. BV was diagnosed using Amsel clinical criteria [Amsel 1983]. Daily vaginal swabs encompassing a 15-day window beginning immediately prior to antimicrobial assay-confirmed initiation of antibiotic treatment were collected spanning 32 individual antibiotic treatment courses from 22 different persons with BV. Nugent scores based on vaginal fluid Gram stain [Nugent 1991] prior to antibiotic treatment were calculated using the baseline vaginal samples to also assess for BV.

### Confirmation of Antibiotic Use

To evaluate adherence to antibiotics, an antimicrobial test was developed to confirm the presence of antibiotics in vaginal swab samples. This assay used vaginal fluid inoculated onto plates with cultured bacteria susceptible to metronidazole. Vaginal swabs were washed by vortex mixing in cold, filtered saline (4°C, Millipore Amicon^®^ Ultra-15 100kDa MWCO Centrifugal Filter) for 2 minutes. Resulting fluid was then centrifuged at 14,000 rpm for 10 min at 4°C to separate pellet. Supernatant was removed and added into holes punctured in agar plates prepared with a lawn of bacteria. 50 μL of supernatant was inoculated onto plates prepared with *Prevotella amnii*, and 100 μL of supernatant was inoculated onto plates prepared with *Megasphaera hutchinsoni*. Presence of antibiotics in the supernatant was indicated by the development of a ring of clearance in the bacterial lawn around the punctured holes in the agar after incubation for 22–24 hours at 37^o^C under anaerobic conditions.

Samples from a treatment course were only included for further analysis if antibiotic presence was observed in at least 80% of samples (a minimum of 4 out of 5 days) if prescribed vaginal metronidazole gel, or 6 out of 7 days if prescribed oral metronidazole starting from the first day of observed presence of antibiotics.

### Measurement of Concentrations of Individual Bacterial Taxa

DNA was extracted from vaginal swabs using QIAamp BiOstic Bacteremia DNA Isolation kit and subjected to taxon-specific 16S rRNA gene qPCR assays to monitor changes in concentrations of thirteen bacterial taxa, each of which has been shown to be associated with elevated HIV infection risk [McClelland 2018, Srinivasan 2018, Srinivasan 2019]. Previously published assays were employed targeting the 16S rRNA gene for *Amygdalobacter indicium* (BVAB2), *Megasphaera lornae, Sneathia* spp. [Fredricks 2009], *Eggerthella-*like sp., *Prevotella amnii* [Srinivasan 2015], *Gemella asaccharolytica, Mycoplasma hominis, Parvimonas* sp. Type 1, *Parvimonas* sp. Type 2, *Porphyromonas* sp. Type 1 [McClelland 2018], Vaginal TM7 [Haggerty 2020], and *Megasphaera hutchinsoni* [Sabo 2023]. A qPCR assay targeting the 16S rRNA gene for *Peptoniphilus lacrimalis* was also developed for this study; the primer/probe sequences and assay reaction conditions are presented in Tables S1 & S2. To test for the presence of PCR inhibitors, an internal amplification control (IAC) PCR assay using jellyfish DNA was conducted on all samples [Khot 2008]. No-template negative controls were included for each qPCR assay.

### Definition of Suppression

For each bacterial taxon, if the concentration of bacterial DNA reached the lower limit of detection for the qPCR assay during the 15-day sampling window, that taxon was defined as “suppressed” for that treatment course. Lower limits of detection ranged from 62.5–187.5 16S rRNA gene copies/swab, varying between taxa on the basis of assay sensitivity. The number of “days until suppression” was counted starting from the baseline day (1 day prior to confirmed antibiotic presence) until the first day for which qPCR data qualified a taxa/treatment course as “suppressed”.

### Data Analysis

To assess significance of observed decreases in concentrations of bacterial DNA from baseline, Student t-tests were conducted for each taxon, using the Holm-Šídák method. Correlation between baseline concentrations of bacterial DNA and time required to suppression was evaluated by linear regression. Observed differences in time to suppression between orally administered vs. topically applied metronidazole gel were assessed for statistical significance by Mann-Whitney U-test. All statistical analyses and data visualization were performed using GraphPad Prism 8.0.

## Results

Thirty-two treatment courses were documented in 22 persons with BV. The mean age of all participants was 31.71 years (SD 6.21). Fifty-five percent of participants self-identified as White, and 41% as Black. All participants self-identified as non-Hispanic. Oral metronidazole was prescribed for 17 total treatment courses, while vaginal metronidazole gel was prescribed for 15. The median Nugent score prior to antibiotic treatment was 8, and scores ranged from 4–10 (Table 1), with clinical diagnosis of BV made by Amsel criteria.

The IAC assay did not detect PCR inhibition in any of the samples, allowing for confidence in the accuracy of subsequent qPCR results. No-template PCR controls were reproducibly negative highlighting the lack of cross-well contamination during qPCR.

**Figure 1.**
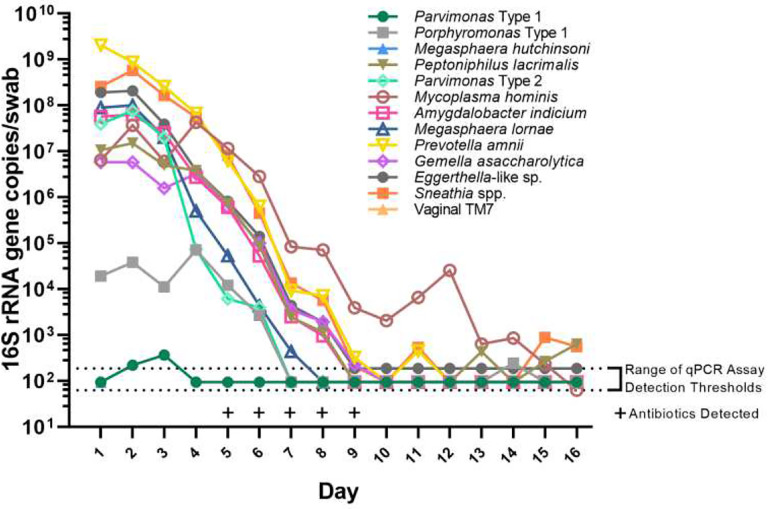
Concentrations of 13 vaginal bacteria over time during metronidazole treatment for a representative treatment course in one person with BV.

**Figure 2.**
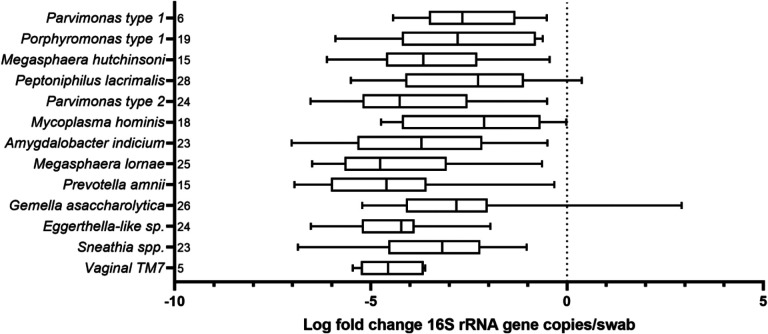
Box plot displaying log-fold change in bacterial concentrations over course of antibiotic treatment for BV, for each of thirteen bacterial taxa. Total number of treatments in which a bacterial taxon appeared is indicated to the right of each bacterial taxon.

**Figure 3.**
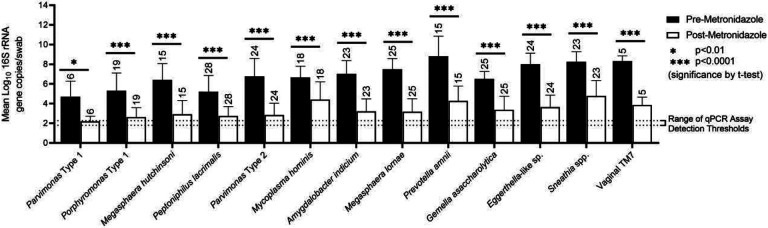
Bar graph showing decreases in concentrations of bacterial DNA from baseline (1 day prior to confirmed antibiotic presence, black bar) to the last day of confirmed antibiotic presence (white bar) for each taxon assayed. Number of treatment courses in which a given taxa was observed is noted above each respective bar. Statistical significance assessed by Student’s t-test.

Mean vaginal bacterial DNA concentrations decreased over the duration of antibiotic administration for all bacterial taxa tested, as reflected by taxon-specific qPCR assays and by documenting use of antibiotics in vaginal fluid by antimicrobial assay (Fig. 1, 2, 3).

Comparison of bacterial DNA concentrations from samples taken before administration of antibiotics to samples taken on the last day of assay-confirmed antibiotic presence showed multiple log10-fold decreases in bacterial DNA across all thirteen taxa. The smallest decrease in bacterial DNA concentration was observed for *Mycoplasma hominis* which only decreased by 2.2 log on average. In the other extreme, we observed an average decrease in *Prevotella amnii* bacterial DNA concentration of 4.5 logs (Fig. 2, 3).

In most cases, bacterial DNA concentrations were reduced to the assay’s limit of detection, suggesting bacterial eradication, though we cannot exclude continued presence of these bacteria at concentrations below our detection threshold. Suppression was noted in all thirteen bacterial taxa monitored, with frequency of suppression varying from 56.5% (*Sneathia* spp., suppression reached in only 13 of 23 treatment courses) to 100% (Vaginal TM7, suppression reached in 5 of 5 treatment courses) (Table 2).

**Figure 4.**
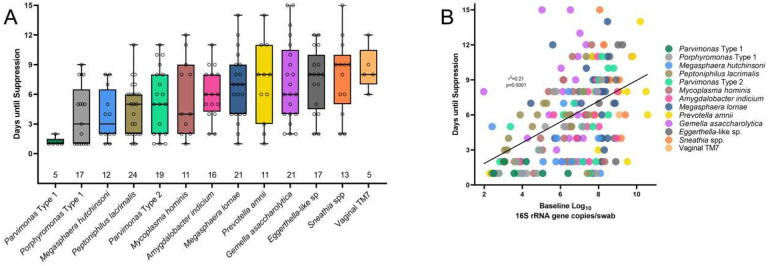
Box plot (A) showing time to suppression for each bacterial taxon (total number of treatments in which a bacterial taxon appeared is indicated along base of x-axis); scatter plot (B) showing relationship between baseline bacterial concentration and time to suppression.

Mean time to suppression varied between taxa. *Parvimonas* Type 1 was one of the most rapidly suppressed taxa, requiring on average only 1.2 days to reach suppression, while vaginal TM7 required the longest time to suppress (average of 8.6 days). Four taxa (*Gemella asaccharolytica, Eggerthella*-like sp., *Sneathia* spp., and Vaginal TM7) required on average greater than 7 days to reach suppression (Fig. 4A). Higher baseline bacterial load was correlated with longer time until suppression (p<0.0001), though the strength of the correlation was low (r^2^=0.21) (Fig. 4B).

**Figure 5.**
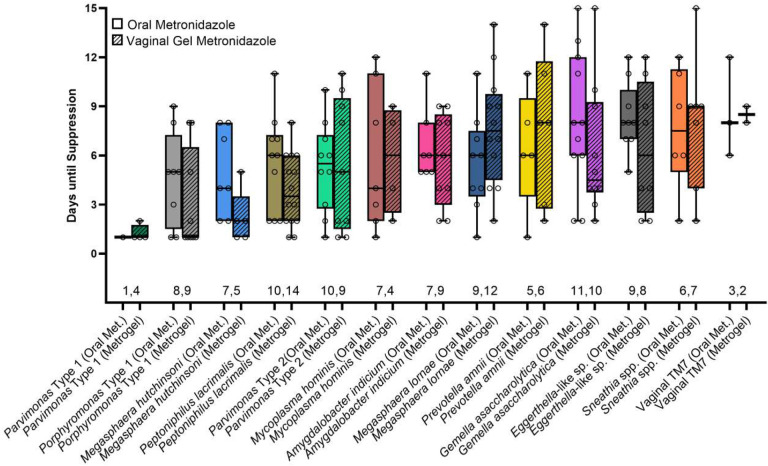
Box plot showing time to suppression between different bacterial taxa, split by method of antibiotic administration (total number of treatments in which a bacterial taxon appeared is indicated along base of x-axis).

No significant differences were observed in time to suppression between orally administered vs. topically applied metronidazole for any of the thirteen assayed taxa (Fig. 5).

## Discussion

In this study, we demonstrate that the administration of metronidazole for treatment of BV reduced concentrations of 13 vaginal bacterial taxa previously associated with increased risk of HIV acquisition as measured by taxon-specific qPCR assays. While all thirteen taxa were found to decrease in concentration over the period of treatment, the magnitude of response of each taxon varied. *Parvimonas* Type 1 and *M. hutchinsoni* were most rapidly depleted, reaching the threshold for suppression in an average of <4 days. In contrast, *G. asaccharolytica, Eggerthella*-like sp., *Sneathia* spp., and Vaginal TM7 were observed to persist for a longer duration, detected above the threshold for suppression for >7 days on average.

Our findings suggest that while metronidazole is effective in reducing the concentrations of bacterial taxa linked to increased risk of HIV acquisition, a 5–7-day treatment course of metronidazole may not be sufficient to fully suppress all taxa, including *Gemella asaccharolytica*, *Eggerthella*-like sp., *Sneathia* spp., and Vaginal TM7. This 5–7-day antibiotic duration has been found effective in treating acute BV. However, the likelihood of recurrence of BV after treatment is high, with observed recurrence rates of up to 80% [Bradshaw 2006, Bradshaw 2016, Vodstrcil 2021]. The exact cause of recurrent BV is not fully understood, and is likely multifactorial, but one potential factor may be the failure of a standard treatment course of metronidazole to completely eradicate all pathogenic bacterial taxa. The results of this study support that hypothesis and indicate that for several vaginal bacterial taxa associated with BV and HIV-acquisition, the standard of care treatment is insufficient for suppression of bacteria to levels undetectable by qPCR. As such, a longer duration of treatment or more bactericidal antibiotics may be required in order to eradicate some vaginal bacteria. Continuation of antibiotic treatment beyond the standard of care has not been observed to be effective at reducing BV recurrence [Schwebke 2007] though initial investigations of high dose vaginal metronidazole therapy have shown encouraging results [Aguin 2014, Sobel 2019].

Metronidazole was observed to be equally effective in suppressing bacterial concentrations when administered orally or as a vaginally applied gel. In a 2009 study, Mitchell et al. compared oral and vaginal metronidazole for the treatment of BV in pregnancy and reported comparable changes in concentrations of most bacterial taxa as evaluated by qPCR [Mitchell 2009]. Notably, *Sneathia* spp. showed greater response to oral treatment, however, we did not note this difference in the present study. This discrepancy may be explained by the low number of individuals with presence of *Sneathia* spp. in our study (vaginal *n*=7).

This study reflects a dense longitudinal analysis of the vaginal microbiota during antibiotic therapy using quantitative PCR to illustrate how several vaginal bacterial taxa associated with increased HIV infection risk respond to metronidazole treatment. The high degree of sensitivity, specificity, and dynamic range for the qPCR assays allow for a high degree of confidence when declaring a bacterial taxon to have been suppressed in a given treatment course. Additionally, the longitudinal nature of the sample set allowed for the capture of data points before, during and following the antibiotic treatment window.

Another strength of this study is the ability to accurately assess antibiotic adherence compared to self-report. An objective test to measure antibiotic presence in a sample allowed us to confirm a participant’s adherence to antibiotic treatment and therefore increase confidence in our conclusions. Additionally, for this study we measured antibiotic response *in vivo*, reflecting biologically relevant concentrations of antibiotic and not requiring *in vitro* cultivation.

A limitation of our study is the modestly sized study population; however, we had sufficient power to demonstrate successful eradication of most bacteria. The longitudinal sample set used in this study was limited to 15 days following the onset of treatment for BV, thus we did not capture the long-term dynamics of the bacterial communities following metronidazole therapy. Furthermore, our definition of suppression does not address the issue of recurrence – it only addresses the capacity for and rate at which metronidazole therapy reduces the concentrations of bacterial taxa to the limits of detection by each respective assay. In terms of the study population, this cohort was comprised of a roughly even split of White and Black persons with a history of BV, located in the Seattle, WA area. As such, the results of this study may not be generalizable to other populations. qPCR measures bacterial DNA concentrations, and the presence of bacterial DNA may not reflect viable bacteria, though the absence of bacterial DNA does imply that concentrations of bacterial cells are low or absent.

We conclude that metronidazole therapy was effective at decreasing vaginal concentrations of thirteen bacterial taxa associated with increased risk of HIV-acquisition, although the time required for suppression often exceeded 7 days for several taxa. Eradication of high-risk vaginal bacteria using metronidazole is one promising avenue to explore for reducing risk for HIV acquisition.

## Supplementary Material

1

## Figures and Tables

**Table 1.** Description of study population.

Characteristic	(n=22)
Age, mean (SD), years	32 (6.2)
Race	
White	12 (54.6%)
Black	9 (40.9%)
Other	1 (4.6%)
Ethnicity	
Hispanic	0 (0%)
Non-Hispanic	22 (100%)
Treatment Courses	(n=32)
Oral Metronidazole (7 d)	17
Vaginal Metronidazole (5 d)	15
Nugent score prior to treatment (median, range)	8 (6)

**Table 2.** Suppression of high-risk bacterial taxa with metronidazole treatment.

Bacterial Taxa	Total Treatments Present, # (%)	Treatments resulting in suppression within 15 days, # (%)
*Parvimonas* Type 1	6 (18.8)	5 (83.3)
*Porphyromonas* Type 1	19 (59.4)	17 (89.5)
*Megasphaera hutchinsoni*	15 (46.9)	12 (80.0)
*Peptoniphilus lacrimalis*	28 (87.5)	24 (85.7)
*Parvimonas* Type 2	24 (75.0)	19 (79.2)
*Mycoplasma hominis*	18 (56.3)	11 (61.1)
*Amygdalobacter indicium*	23 (71.9)	16 (69.6)
*Megasphaera lornae*	25 (78.1)	21 (84.0)
*Prevotella amnii*	15 (46.9)	11 (73.3)
*Gemella asaccharolytica*	26 (81.3)	21 (84.0)
*Eggerthella*-like sp.	24 (75.0)	17 (70.8)
*Sneathia* spp.	23 (71.9)	13 (56.5)
Vaginal TM7	5 (15.6)	5 (100.0)
