# Supplementary material for: Effect of Metronidazole on Concentrations of Vaginal Bacteria Associated with Risk of HIV Acquisition
Source: Res Sq. 2024 Apr 11:rs.3.rs-4219764. Preprint. [Version 1] doi: 10.21203/rs.3.rs-4219764/v1 (PMC11042432; doi:10.21203/rs.3.rs-4219764/v1)
Supplement: 1 [file NIHPPrs4219764V1-supplement-1.pdf]

Supplemental Materials

Table S1. *P. lacrimalis* qPCR assay Primer/Probe sequences and reaction conditions.

PCR Assay	PCR Conditions	Amplicon Size (bp)	Primer/probe	Primer/probe sequence	Sensitivity (no. of gene copies/reaction)
Peptoniphilus lacrimalis	50°C Uracil-N-glycosylase incubation, 2 min<br>95°C Taq Activation, 10 min<br><br>45 cycles<br>95°C Melt, 15 sec<br>57°C Anneal, 39 sec<br>72°C Extend, 30 sec	150	Placrimalis_989F<br>Placrimalis_1139R<br>Placrimalis_1017-1044_pb	5'-GCTTGACATATAAGAGACGAACT-3'<br>5'-CCGAAATGCTGGTAACTAGTAA-3'<br>5'-FAM-TAAGTTTTCTTCTTCGGAAGCCCTTATA-TAMRA-3'	2.5

Table S2. *P. lacrimalis* qPCR master mix composition.

PCR Reagent	Final reaction concentration
qPCR Buffer A	1 mM
$MgCl_2$	3 mM
dNTP mix *with* dUTP	1 mM
Placrimalis_989F	0.8 uM
Placrimalis_1139R	0.8 uM
Placrimalis_1017-1044_pb	150 uM
AmpErase ® Uracil N-glycosylase (UNG)	0.001 U
AmpliTaq ® Gold DNA Polymerase	0.02 U
